# Applicability of a canine prostate simulator (PROSIM-DOG) in clinical veterinary practices

**DOI:** 10.3389/fvets.2025.1631989

**Published:** 2025-10-13

**Authors:** Elvira Matilla-Pinto, Carolina Balao da Silva

**Affiliations:** VALORIZA - Research Centre for Endogenous Resource Valorization, Portalegre Polytechnic University, Portalegre, Portugal

**Keywords:** DOG, accessory sex gland, theriogenology, veterinary technician, training device, transrectal digital palpation

## Abstract

Simulators help students to perform procedures as many times as needed, in a controlled and non-stressful environment. It is crucial to include transrectal digital palpation in the physical examination of dogs for effective clinical diagnosis of prostate diseases, since this is a commonly found pathology in intact males. Training in prostate palpation may be hindered if students cannot assess and compare different types of prostates, a common situation that can be solved by training devices. A low-cost canine prostate simulator (PROSIM-DOG) was developed and tested by 167 veterinary science students, in order to compare their perspective and usefulness of the simulator. Students were divided into four groups with varying levels of instructional support. Group 1 (*n* = 35) received a technique description, used the simulator, and completed two questionnaires (simulator and real examination). Group 2 (*n* = 35) received a description, visual aids, and palpated inert prostates without the simulator before the live exam. Group 3 (*n* = 36) received only a written description before the live exam. Group 4 (*n* = 61) had access to a description, inert prostates, the simulator, and completed the questionnaire to assess device applicability. Additionally, fourteen faculty members also answered a third questionnaire, in order to validate PROSIM-DOG. Students with access to images and simulator palpation (group 2) achieved higher diagnostic accuracy (66.6%), while most in the description-only group (group 3) reported uncertainty during examination (80%) and considered the teaching method insufficient (77.7%). Overall, all students positively evaluated the device and considered that it significantly improved their understanding of canine prostatic anatomy and pathology, increased confidence to perform transrectal digital palpation in dogs and that it reduced their anxiety associated with first-time clinical procedures. Enquired faculty members endorsed the simulator's educational value, emphasizing its potential to standardize practical training. Fourteen faculty members also answered a third questionnaire, endorsed the simulator's educational value, emphasizing its potential to standardize practical training. These findings support the integration of *PROSIM-DOG* into veterinary curricula as a valuable tool for both preclinical preparation practices and skill reinforcement in clinical rotations. Future research should focus on longitudinal studies assessing the impact of simulators on student performance and diagnostic accuracy in real clinical contexts.

## 1 Introduction

Assisted repetitive practices and adding simulators to veterinary teaching methodologies is becoming a reality to universities, helping students to perform the procedures as many times as needed, in a controlled and non-stressful environment (1). Animal reproduction is one of the areas where simulators have had a huge development, although the cost of devices usually consists of a limitation to its acquisition. Prostate diseases are frequently seen in adult dogs, particularly in elderly ones. Among prostatic canine diseases, Benign Prostatic Hyperplasia (BPH) is the most commonly found pathology, along with prostatitis, prostatic cysts and prostatic neoplasia (2). Clinical signs usually common to all prostatic diseases in dogs are: lower urinary tract symptoms, seminal alterations, gastrointestinal symptoms, locomotor disorders and systemic signs (3).

It is crucial to include transrectal digital palpation in the physical examination of dogs for effective clinical diagnosis, which can be complemented with other examinations such as radiography, ultrasonography, cytology and microbiology of prostatic, urethral and seminal fluid, prostatic aspiration or biopsy (4, 5).

The technique of prostate palpation in dogs is carried out performing simultaneous abdominal and transrectal digital palpation: using one hand to palpate the caudal abdomen and push the neck of the bladder and prostate into the pelvic canal; and introducing a finger from the other hand into the rectum and accurately assess the prostate's location, size, symmetry, surface contour, movability, consistency and pain (4). The normal prostate is smooth, symmetrical, movable and nonpainful on palpation, with an easily palpable dorsal median groove (2–4).

Training in prostate palpation may be hindered if students cannot assess and compare different types of prostates, a common situation in classrooms. Furthermore, the fact that the palpation cannot be done simultaneously by student and teacher is a limitation to the hands-on methodology.

Therefore, homemade devices such as PROSIM-DOG are a low-cost alternative that educators can develop and apply according to their needs, performing adjustments whenever required. Clinical teaching is a complex learning situation influenced by the learning content, the setting and the participants‘ actions and interactions. Pedagogical strategies encompass different focus in teaching, either a focus on the teacher's knowledge and behavior or the student's behavior and understanding (6). Different methodologies have also been applied to clinical teaching, to improve learning outcomes and competences acquisition, in nursing students (7) and also in veterinary students (8), Overall, these teaching strategies aim to improve students' preparation in order to increase their ability to adapt and cope with the requirements of eventual workplaces upon graduation (9).

Several studies have addressed mental health issues in veterinary medicine students, emphasizing out elevated levels of academic stress as well as transitional stress in junior students, both related with depression and anxiety symptoms (10), an enhanced sense of hopelessness in final-year students (11) or an increase in the amount of students seeking professional counseling on aspects related to their mental health (12). It is therefore important that veterinary education institutions implement strategies to mitigate these stressors and support students' wellbeing. One such strategy is the development and integration of simulation-based learning tools, which can enhance clinical skills acquisition in a controlled and low-risk environment (13).

The main objective of this work was to assess the teaching materials provided to the students, as well as the PROSIM-DOG simulator and the effectiveness of this methodology by students themselves and teachers with expertise in clinical areas.

## 2 Materials

### 2.1 Simulator assemblance

**Figure 1:**
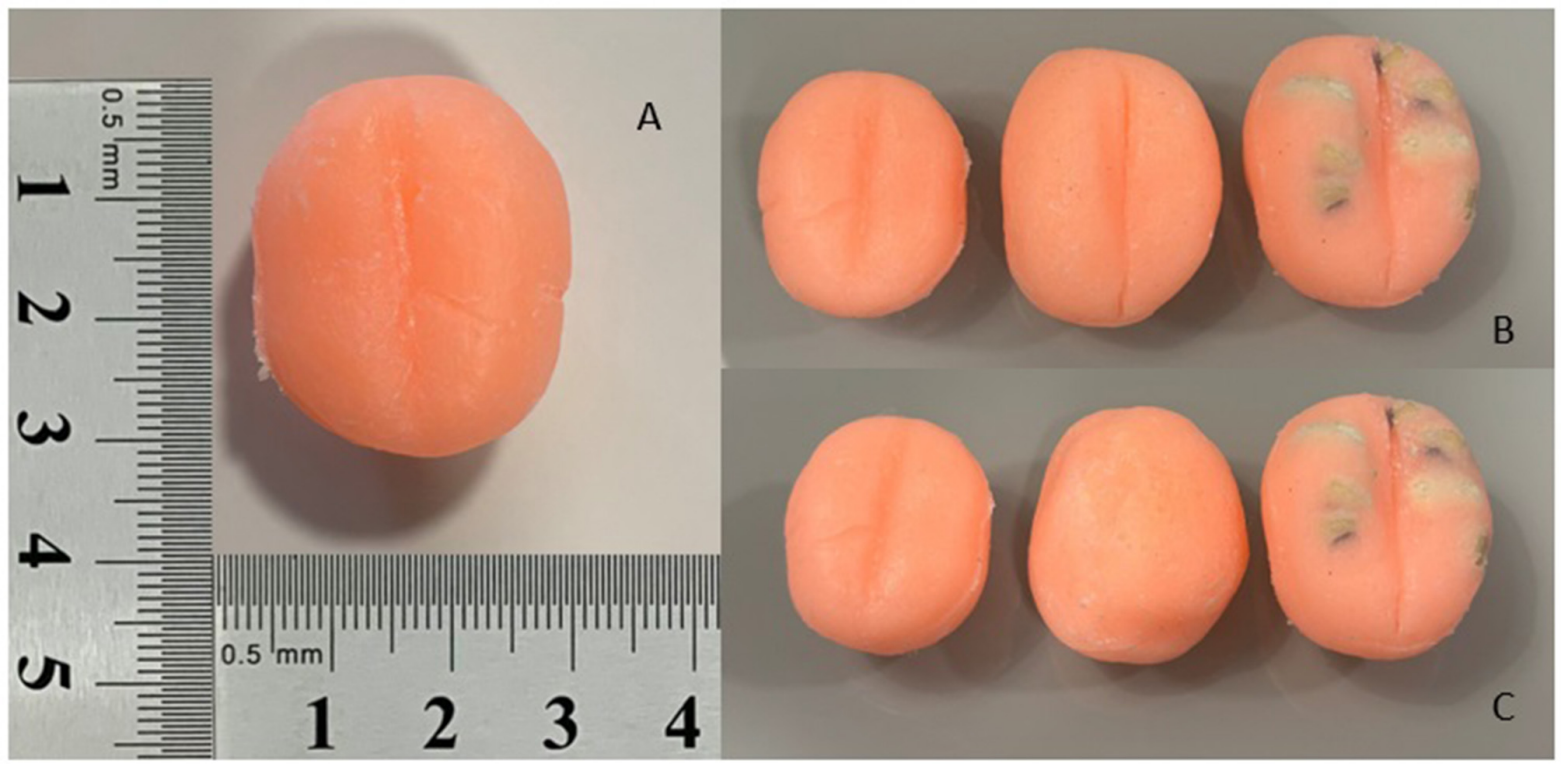
Silicone canine prostates modeled in three different sizes and forms: **(A)** normal prostate from a medium-sized dog; **(B)** normal, hyperplastic, and hyperplastic with tumors (left to right); **(C)** normal, hyperplastic with loss of lobular definition, and hyperplastic with tumors (left to right).

**Figure 2:**
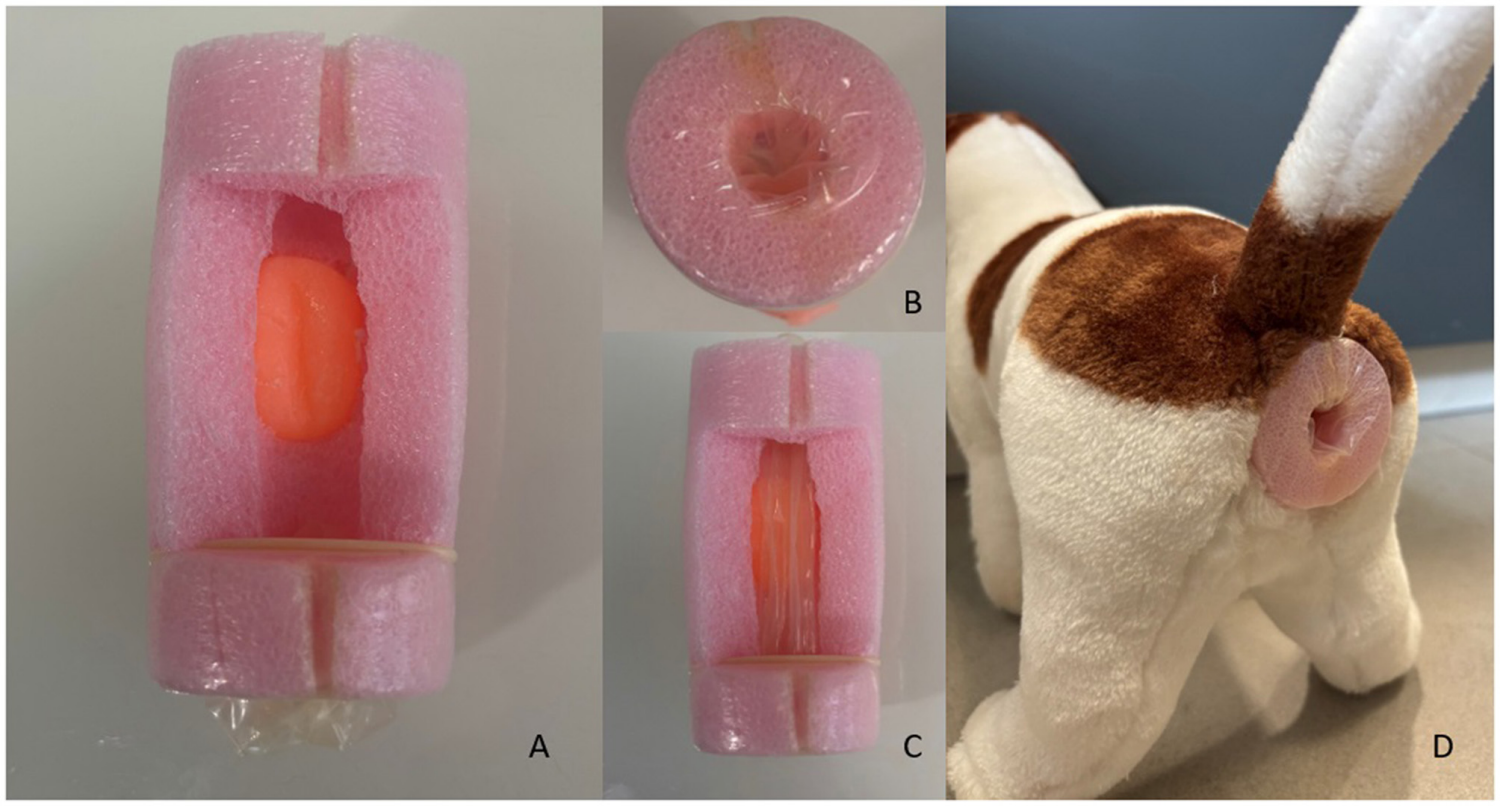
**(A)** Polyethylene cylinder with a ventral opening for exchanging different prostates and a dorsal window. **(B)** A thin layer of latex covering the terminal end of the cylinder. **(C)** Simulation of the rectum using the latex membrane. **(D)** Medium-sized stuffed dog holding the device.

A low-cost device (PROSIM-DOG) was developed using a polyethylene-based structure, consisting of a firm but deformable cylinder with a hole positioned on the inferior side for exchanging rigid structures simulating different prostates. Prostates were formed with silicone modeled in three different sizes and forms: normal, hyperplastic, and hyperplastic with tumors. The hyperplastic prostate can be inverted to simulate a hyperplastic prostate with loss of lobular definition (Figure 1). The silicone prostates were introduced through the ventral cavity previously made on the polyethylene cylinder. The cylinder has a dorsal window to provide clearance and allow for digital palpation. A thin layer of latex was placed to cover the terminal end of the cylinder and simulate the rectum. The device was introduced in a medium size stuffed dog at the approximate anatomic location of the rectum (Figure 2).

## 3 Methods

### 3.1 Study design

A total of 167 veterinary science students from Portalegre Polytechnic University and University of Évora, Portugal and from Extremadura University, Spain, were divided into 4 study groups. In Évora and Extremadura University, enquired students were all enrolled in Veterinary Medicine (group 4), while in Portalegre Polytechnic University questionnaires were only delivered to Veterinary Nursing students (groups 1, 2 and 3). The first group (group 1; *n* = 35) was provided with a description of the technique, the simulator, and an evaluation questionnaire for the simulator (questionnaire 1). After the experience, these students performed a real examination on a dog and filled out another questionnaire about the real experience (questionnaire 2). The second group (group 2; *n* = 35) was shown a sheet with images and a description of the technique, and they were introduced to normal, hyperplastic, and neoplastic silicone-inert prostates that they could palpate individually. Afterwards, students performed an *in vivo* examination and evaluated its condition, fulfilling questionnaire 2. The third group (*n* = 36) was only provided with a verbal description of the technique, lacking either visual support or simulator. These students also performed an *in vivo* examination and were required to evaluate the prostatic condition, further answering questionnaire 2. Students from group 4 (*n* = 61) were provided with a verbal description of the technique, individual palpation of different inert prostates (normal, hyperplastic and neoplastic) and could also palpate these prostates inserted on the simulator. Finally, students from group 4 also answered questionnaire 1. The simulator was additionally evaluated by academic personnel with clinical experience, who completed questionnaire 3 as part of the validation process for *PROSIM-DOG*. Questionnaires 1, 2 and 3 were conducted in accordance with the recommendations of the Ethics Committee of the Portalegre Polytechnic University and can be found, respectively, in Supplementary Appendixes 1–3.

#### 3.1.1 Questionnaires

Questionnaire 1 was structured as an anonymous questionnaire to enquire veterinary science students from the first to the fourth year. This questionnaire comprised 14 closed-ended questions designed to assess prior knowledge of canine prostatic pathologies, previous practical experience with prostatic examinations, and perceptions regarding the use of simulators. Students were also asked to evaluate the similarity between simulator-based training and real-life procedures, as well as their confidence levels before and after simulator use. Additionally, questions explored students' feelings of pressure during live animal practice and their opinions on the usefulness and areas of improvement for veterinary simulators. Questionnaire 1 can be consulted in Supplementary Appendix 1.

Questionnaire 2 included four close-ended questions, and it is included in Supplementary Appendix 2. The purpose of the first question was to assess whether the student was able to correctly identify the condition of the prostate in a live dog. Furthermore, on a second question students were asked to evaluate whether the educational materials provided prior to the practical session—including theoretical content, visual aids, and simulator-based training—were adequate to make them able to perform the technique confidently and accurately. This questionnaire also encouraged students to comment on improvement areas for the practical classes in the area of animal reproduction including two questions directly related to it.

To evaluate the pedagogical effectiveness of PROSIM-DOG, questionnaire 3 was presented to academic staff working in clinical area, summing a total of 14 answers (*n* = 14). The instrument consisted of nine questions, including seven multiple-choice items and two open-ended questions. The multiple-choice items assessed general user experience, the simulator's usefulness for teaching palpation techniques, clarity in anatomical representation, tactile realism, depiction of both healthy and pathological prostates, ease of use during practical sessions, and its perceived impact on students' skill development. The open-ended questions invited suggestions for improvement and additional comments. Questionnaire 3 can be found in Supplementary Appendix 3. Participation was voluntary and anonymous, and all responses were treated with strict confidentiality.

## 4 Results

**Figure 3:**
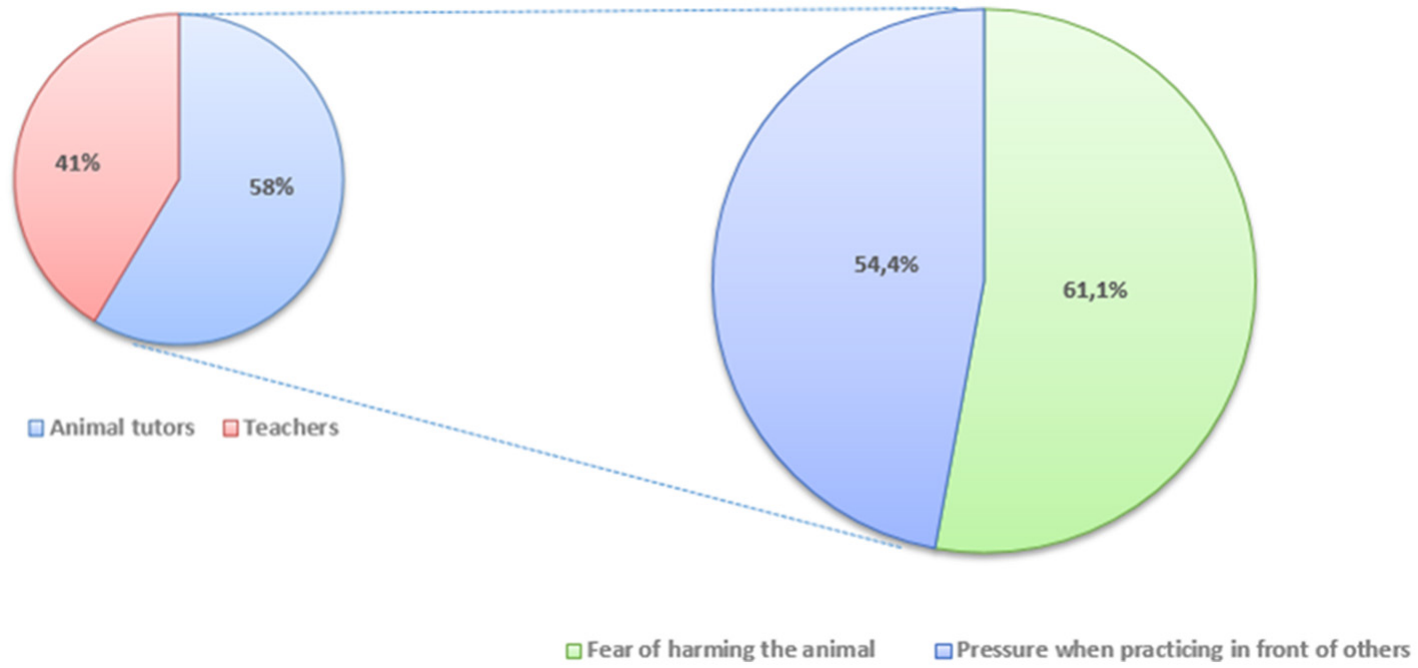
Distribution of veterinary science students by study group and completed questionnaires. Group 1-Veterinary Nursing (VN) (*n* = 35) completed both questionnaire 1 and 2; Group 2-VN (*n* = 35) and Group 3-VN (*n* = 36) completed only questionnaire 2; Group 4-Veterinary Medicine (VM) (*n* = 61) completed only questionnaire 1. Percentages in the pie chart represent the proportion of total responses per questionnaire.

A total of 167 veterinary science students were divided into four study groups as previously described. Despite groups 1, 2 and 3 consisted exclusively of veterinary nursing students, and group 4 comprised veterinary medicine students, questionnaire responses were grouped, since the main goal was to assess simulator applicability in general clinical practices (Figure 3). Most students had limited prior experience in prostatic palpation. Specifically, 5 students reported not having taken the reproduction course, with no previous experience or knowledge of prostatic pathologies. Although 18 students had completed the course, only two of them reported prior practical experience; their self-reported level of knowledge was medium. The remaining students were currently enrolled in the course; and from these only four had performed a previous prostatic palpation and all reported having low knowledge of prostatic diseases.

**Figure 4:**
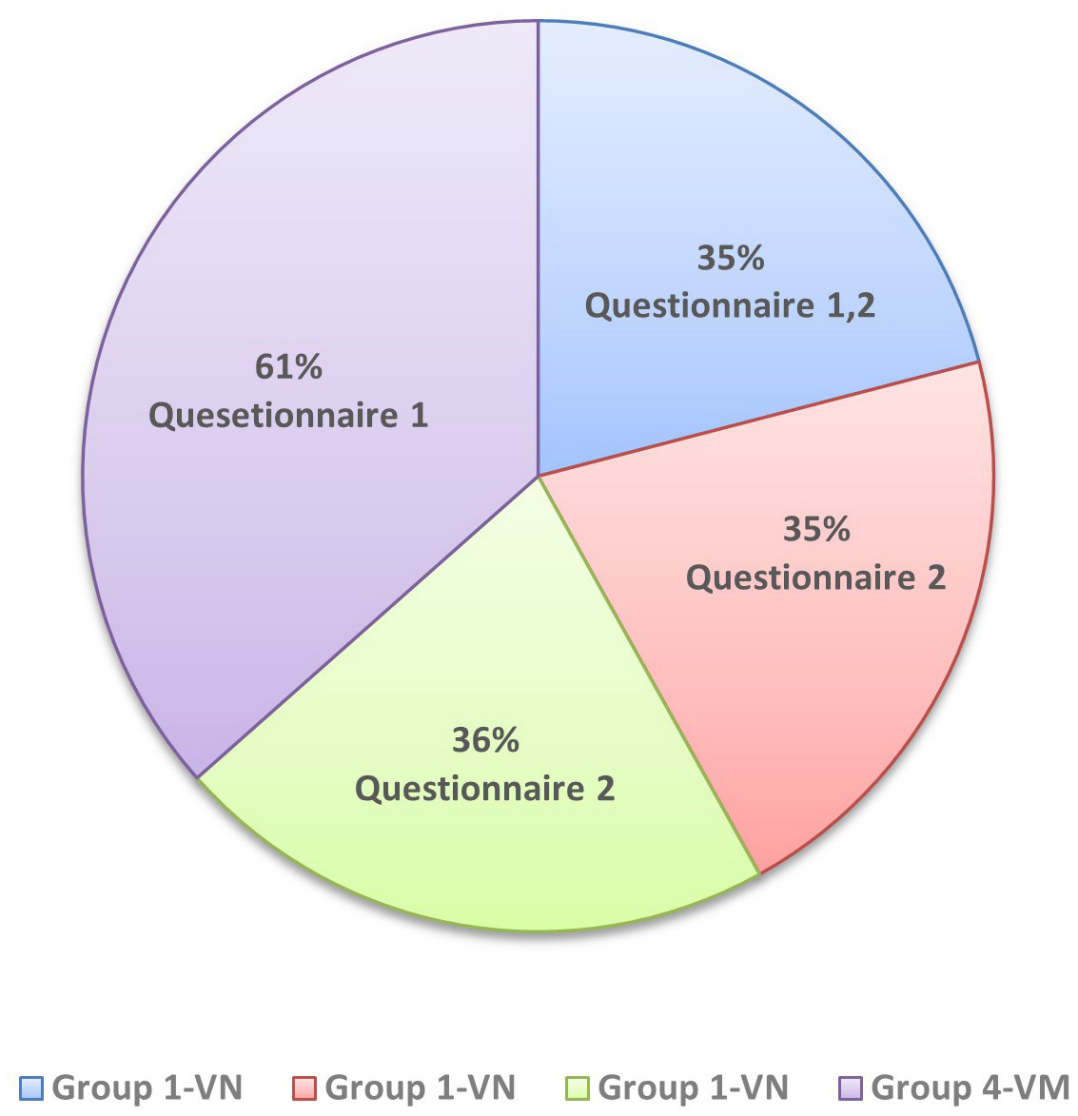
Students' emotional responses: 61.1% admitted feeling fear of harming the animal during live practice, and 54.4% reported feeling pressure when practicing in front of others, 58% feeling mostly intimidated by the presence of animal tutors, while 41% felt pressured by the presence of teachers.

Regarding students' emotional responses, 61.1% admitted feeling fear of harming the animal during live practice, and 54.4% reported feeling pressure when practicing in front of others. The main source of pressure was the presence of animal tutors (58%), followed by the presence of teachers (41%) (Figure 4).

As stated before, only groups 1, 2 and 3 could perform transrectal digital palpation in a dog, after previously testing the simulator with different prostates (group 1, *n* = 35); checking images and palpating inert prostates (group 2, *n* = 35); or being provided solely a description of the technic, with no access to simulator or inert prostates (group 3, *n* = 36). In group 1, a total of 78.6% of the students correctly identified the prostate condition during live examination, while in group 2 only 66.6% were able to indicate correctly this condition. In group 3, 80% of the respondents admitted not knowing whether they had palpated the prostate during the live examination. Furthermore, 77.7% of students in this group considered that the teaching methods were insufficient and highlighted that additional resources such as images or videos would facilitate their learning.

**Figure 5:**
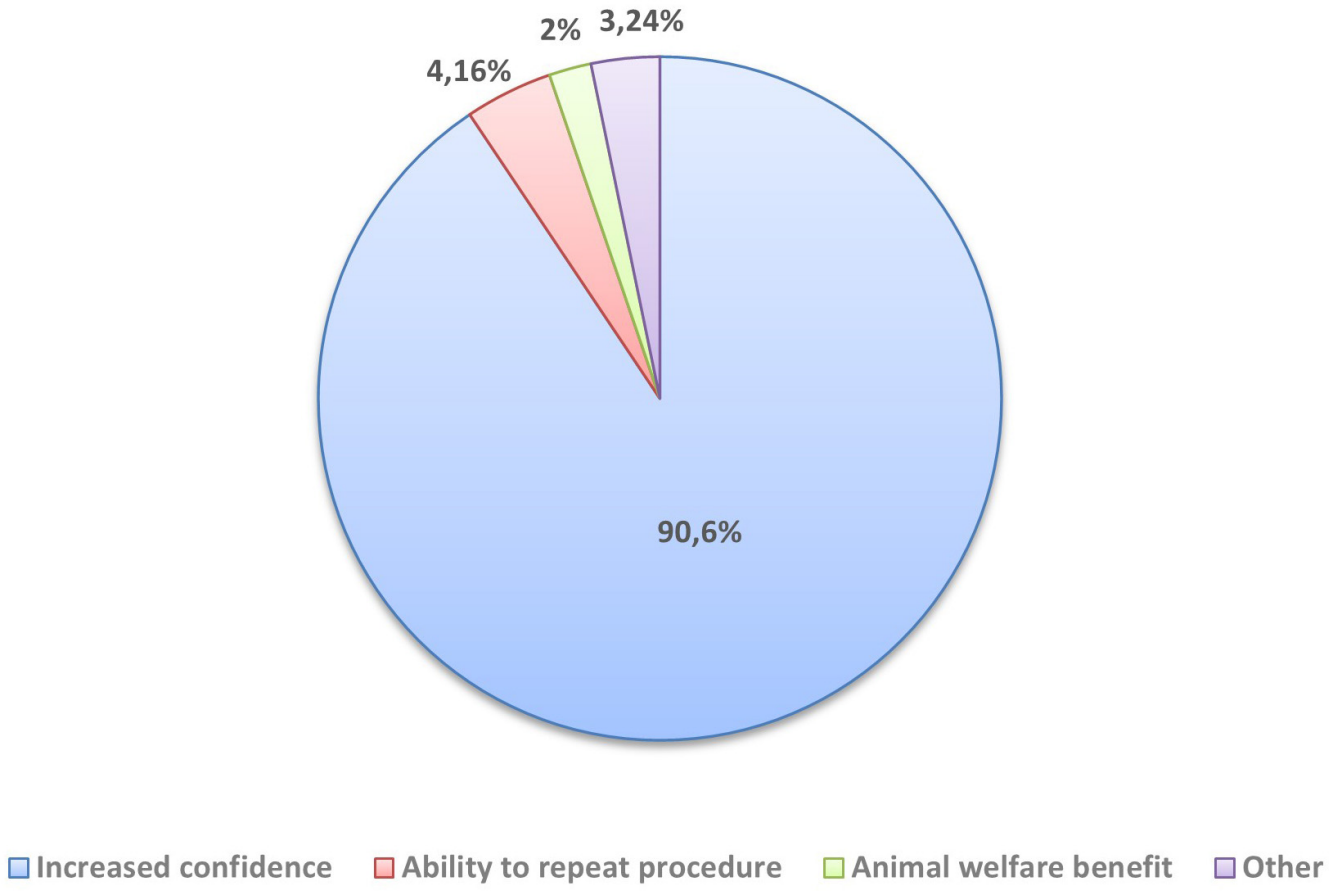
Perceived benefits of using the PROSIM-DOG simulator by students from Groups 1 and 4 (*n* = 96).

All 96 students from group 1 and group 4 who were able to test the simulator PROSIM-DOG rated it as 100% useful in the second questionnaire. The majority (90.6%) indicated that the main benefit of the simulator is the fact that it increases one's confidence prior to performing the technique on live animals. Additionally, 4.16% of the respondents highlighted the possibility of repeating the procedure multiple times as its main advantage, while only 2% of them valued its contribution to animal welfare by reducing the need to use live animals in teaching (Figure 5).

**Figure 6:**
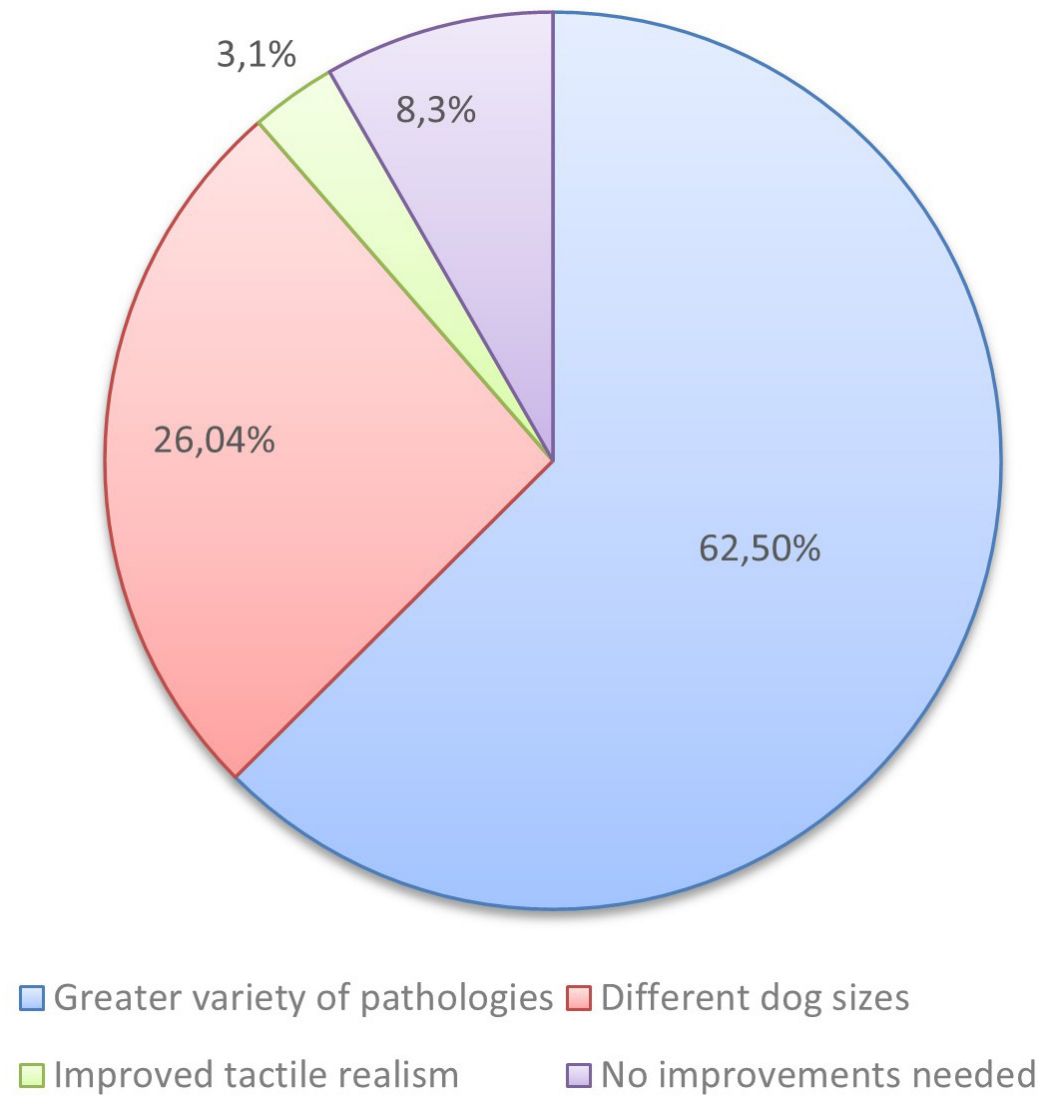
Distribution of responses regarding suggested improvements to the prostatic palpation simulator from groups 1 and 4 (*n* = 96). The majority of participants indicated a preference for a greater variety of pathological conditions (62.5%), followed by different dog sizes (26.04%). A smaller proportion of respondents suggested improved tactile realism (3.1%), while 8.3% considered no improvements necessary.

After analyzing the answers to possible improvements suggested to the simulator, 26.04% of the students recommended the inclusion of a wider range of dog sizes, 62.5% proposed adding a greater variety of pathological conditions, 3.1% suggested improving tactile realism, and 8.3% stated that no improvements were necessary (Figure 6).

Regarding questionnaire 3, all participating educators (*n* = 14) positively evaluated PROSIM-DOG, unanimously considering it to be an effective pedagogical tool. Respondents agreed that the simulator contributes to improving students' practical skills in prostate palpation and facilitates a better understanding of prostatic anatomy. The simulator was also rated as easy to use during practical sessions. Regarding tactile realism, nine out of 14 respondents classified the texture as “realistic,” while five out of 14 considered it “very realistic.” One suggestion for improvement, highlighted in the open-ended responses, was the inclusion of simulated feces within the rectum, to better mimic clinical scenarios where stool may interfere with palpation.

## 5 Discussion

The canine prostate is often affected by several pathologies, especially in unsterilized males older than 6 years old. Some of this diseases frequently share the same clinical signs, which can hinder a correct diagnosis (2). Therefore, improving diagnostic techniques of prostatic disease in dogs such as transrectal digital palpation is crucial in order to promote early recognition and adequate treatment of the disease. Despite this clinical procedure is not among the most invasive, it still represents a disruption to the wellbeing of animals. This sense of causing discomfort to the animal can surely represent a setback in the students and interfere with their training. The level of preparedness, which can be defined as a measure of the likelihood that the veterinary student is going to be a capable learner and participant during workplace clinical training (8), directly influences student performance. Therefore, enhancing preparedness prior to workplace clinical training is critical for both educational outcomes and student wellbeing. In our work, a total of 61.1% of students reported feeling afraid of causing harm to the animal during live practice, while 54.4% felt pressure when performing in front of others. The majority (90.6%) identified increased confidence prior to performing the technique on live animals as the simulator's main benefit.

It is well established that the principle of the three Rs (reduction, refinement, and replacement), defined by Russell and Burch (14), guides the use of animals in scientific research. However, these principles may not be entirely transferable to veterinary education, as their philosophical and scientific foundations were originally intended for conducting and validating experimental procedures rather than for the development of manual clinical skills. Moreover, a lack of hands-on practice in real-life settings may adversely affect the quality of veterinary training (15). Animal welfare has been identified as a key priority within the third Strategic Plan of the World Organization for Animal Health (16), further supporting the increasing adoption of simulation models in veterinary medicine. Although the use of live animals during practical training remains possible, the opportunity to compare healthy conditions with pathological states is extremely limited. Additionally, the inability for both student and instructor to palpate simultaneously presents a significant limitation of traditional hands-on methodologies. Effective training programmes require direct and repetitive application of techniques to ensure skill acquisition (23). In our study, a low percentage of students (4.16%) highlighted the possibility of repeating the procedure multiple times as the main advantage. Moreover, only 2% of the respondents identified the protection of animal welfare as the primary advantage of using simulators in veterinary sciences, which may be attributed to the fact that the tested procedure—transrectal digital palpation—is not considered a highly invasive clinical intervention.

The simulator was unanimously rated as useful by students and clinical professionals, nevertheless, both suggested several pertinent improvements to enhance the purpose of the simulator, such as adding simulated feces in the rectum to better mimic clinical conditions, incorporating a wider variety of pathological cases and including different dog sizes to reflect anatomical diversity. Additionally, instructors also highlighted the realism and educational effectiveness of PROSIM-DOG. Numerous studies have demonstrated the effectiveness of veterinary simulation techniques, showing significant improvements in clinical skills and reductions in training time through structured simulator-based programs (17), offering advantages in animal welfare and out-of-school training, as well as financial saving benefits (18). Previous research has also demonstrated that canine prostate palpation simulators can successfully aid students in developing the clinical skills necessary for accurate prostate examination in dogs (19). Overall, by bridging the gap between theoretical knowledge and real-world application, simulators can significantly contribute to the development of core competencies expected in clinical settings (24).

This study highlights the positive impact of combining theoretical instruction with simulation-based training in enhancing veterinary students' ability to perform prostatic palpation and accurately identify prostatic conditions in live animals. The sense of touch represents a fundamental dimension due to its role at the neuro-motor and cognitive level (20). After evaluating the responses of veterinary nursing students who received only a verbal description without visual or simulator support (group 3), we verified that 80% of the respondents admitted they were uncertain whether they had palpated the prostate during the live examination. Moreover, 77.7% of these students (group 3) felt that the teaching methods were insufficient and emphasized that additional resources, such as images or videos, would help improve their learning experience.

The results of this study reveal a general lack of prior experience and limited knowledge of canine prostatic diseases among veterinary students (group 4), coupled with high levels of anxiety and perceived pressure during live animal practice (on all groups). These findings underline the value of simulators not only as technical training tools but also as resources to reduce stress and promote more ethical and motivational teaching approaches. Educational theory reinforces that learners are motivated by their own interests and must be aware of objectives and assessment criteria in order to take responsibility for their progress (21). Learning is context-dependent, and knowledge is most effectively acquired and applied in real clinical settings, making early and appropriate clinical exposure essential.

The development of advanced simulators is essential to support educators in complying with modern ethical guidelines, which prioritize the protection of animal welfare and the reduction of reliance on live animals for teaching and pedagogical research purposes (22). The universal recognition of the simulator's usefulness by students, along with students' suggestions for improvements—such as the inclusion of additional pathological variations and models representing different animal sizes—strongly supports its continued refinement and implementation. Overall, the results of this article focused on PROSIM-DOG emphasize the importance of incorporating high-fidelity simulation and diverse teaching methodologies in veterinary education to improve practical skills, enhance student confidence, and reduce dependence on live animals during early stages of training. All educators evaluated the prostate palpation simulator positively, unanimously recognizing it as an effective teaching tool that enhances students' practical skills and anatomical understanding. Further longitudinal studies are recommended in order to improve PROSIM-DOG and other similar devices, adjusting it to the needs of the students and improving the transfer of knowledge in veterinary sciences, while coping with animal welfare issues.

## Data Availability

The raw data supporting the conclusions of this article will be made available by the authors, without undue reservation.
