# Supplementary material for: Applicability of a canine prostate simulator (PROSIM-DOG) in clinical veterinary practices
Source: Front Vet Sci. 2025 Oct 13;12:1631989. doi: 10.3389/fvets.2025.1631989 (PMC12557791; doi:10.3389/fvets.2025.1631989)
Supplement: Supplementary file 1 [file Data_Sheet_1.PDF]

POLITÉCNICO DE PORTALEGRE
Escola Superior de Biociências de Elvas

Questionnaire 1 – Prostatic training with PROSIM-DOG

This questionnaire forms part of a research study conducted by Professors Elvira Matilla Pinto and Carolina Balão da Silva, affiliated with the Biosciences Superior School of Elvas – Portalegre Polytechnic University. The purpose of this study is to evaluate the perceptions of Veterinary Medicine and Veterinary Nursing students regarding the use of the PROSIM-DOG prostatic simulator in their academic training

Data collected will be used exclusively for academic and scientific purposes. Participant anonymity and data confidentiality will be strictly maintained in accordance with ethical research guidelines. No personally identifiable information will be recorded or disclosed.

There are no correct or incorrect responses. Participants are encouraged to respond sincerely and spontaneously to each item by marking an "X" in the appropriate box. Responses should reflect personal perceptions based on the use of the PROSIM-DOG simulator.

Thus, having been properly informed and clarified, I voluntarily agree to participate in this study/project. ☐

1. **Current year of enrollment in the degree program:**
   ☐ First
   ☐ Second
   ☐ Third
   ☐ Fourth
   ☐ Fifth

2. **Have you completed the course on Reproduction and Obstetrics?**
   ☐ Yes
   ☐ No

3. **How would you rate your knowledge regarding the clinical relevance of prostatic disorders in dogs?**
   ☐ High
   ☐ Moderate
   ☐ Low
   ☐ None

4. **Have you ever performed a prostatic examination?**
   ☐ Yes
   ☐ No

5. **If you answered "Yes" to the previous question, how many times have you performed the technique?**
   ☐ Once
   ☐ Fewer than 5 times
   ☐ 5–20 times
   ☐ More than 20 times

6. **If you answered "Yes" to question 4, how would you assess your experience using the simulator?**
   ☐ Identical to real-life practice
   ☐ Very similar
   ☐ Fairly similar
   ☐ Slightly similar
   ☐ Not similar at all

7. **Do you feel pressured when performing procedures on live animals in front of others?**
   (You may select more than one option)
   ☐ Instructors
   ☐ Animal caretakers
   ☐ Fellow students
   ☐ Other professionals
   ☐ None

8. **Do you experience fear of harming the animal, making you more nervous during the procedure?**
   ☐ Yes
   ☐ No

9. **Do you experience fear of failure, preferring not to perform the procedure and allowing others to do it instead?**
   ☐ Yes
   ☐ No

10. **After training with the simulator, do you feel more confident about performing diagnostics on live animals?**
    ☐ Yes
    ☐ No
    ☐ Not sure

11. **Have you previously worked with other simulators during veterinary training?**
    ☐ Yes
    ☐ No

12. **Do you believe that the use of simulators is helpful before performing certain procedures on live animals?**
    ☐ Yes
    ☐ No

13. **In your opinion, what is the main benefit of using simulators?**
    ☐ Gaining confidence before live animal practice
    ☐ Promoting animal welfare by avoiding the use of live animals
    ☐ Ability to repeat techniques multiple times
    ☐ I do not believe simulators are beneficial
    ☐ Other: ____________

14. **How would you improve the simulator you have used? (You may select more than one option)**
    ☐ Improved tactile feedback
    ☐ Availability of different animal sizes
    ☐ Inclusion of additional prostatic pathologies
    ☐ No improvements needed
    ☐ Other: ____

The authors gratefully acknowledge your contribution to this research.
