# Supplementary material for: Applicability of a canine prostate simulator (PROSIM-DOG) in clinical veterinary practices
Source: Front Vet Sci. 2025 Oct 13;12:1631989. doi: 10.3389/fvets.2025.1631989 (PMC12557791; doi:10.3389/fvets.2025.1631989)
Supplement: Supplementary file 2 [file Data_Sheet_2.PDF]

Questionnaire 2 – Live animal evaluation

This questionnaire forms part of a research study conducted by Professors Elvira Matilla Pinto and Carolina Balão da Silva, affiliated with the Biosciences Superior School of Elvas – Portalegre Polytechnic University. The purpose of this study is to evaluate the perceptions of Veterinary Medicine and Veterinary Nursing students regarding the use of the PROSIM-DOG prostatic simulator in their academic training

Data collected will be used exclusively for academic and scientific purposes. Participant anonymity and data confidentiality will be strictly maintained in accordance with ethical research guidelines. No personally identifiable information will be recorded or disclosed.

There are no correct or incorrect responses. Participants are encouraged to respond sincerely and spontaneously to each item by marking an “X” in the appropriate box. Responses should reflect personal perceptions based on the use of the PROSIM-DOG simulator.

Thus, having been properly informed and clarified, I voluntarily agree to participate in this study/project. ☐

1. **When palpating a live animal, I consider the prostate to be:**

   ☐ I am not sure whether I evaluated the prostate

   ☐ Normal

   ☐ Hyperplastic

   ☐ Neoplastic

   ☐ I am unsure about the size of the prostate

2. **I believe the instructional materials provided were sufficient to perform the prostatic examination:** *(Mark one or more options with an “X”)*

   ☐ Yes

   ☐ No

   ☐ I consider it essential to perform the procedure on a live animal

   ☐ Additional visual support is needed (e.g., videos, images)

   ☐ Practical training with ex vivo organs or an inert model is necessary

3. How effective do you find the current structure of the practical classes?

- ☐ Very effective – I learn a lot and feel well-prepared
- ☐ Generally effective – minor improvements could be made
- ☐ Neutral – the classes are fine but not outstanding
- ☐ Ineffective – I struggle to understand the material
- ☐ Very ineffective – the sessions need major changes

2. Which area of the practical classes do you think could benefit most from improvement?

- ☐ Clarity of instructions and demonstrations
- ☐ Availability of materials and equipment
- ☐ Time management during sessions
- ☐ Relevance of activities to theoretical content
- ☐ Opportunities for hands-on practice

The authors gratefully acknowledge your contribution to this research.
