# Supplementary material for: Applicability of a canine prostate simulator (PROSIM-DOG) in clinical veterinary practices
Source: Front Vet Sci. 2025 Oct 13;12:1631989. doi: 10.3389/fvets.2025.1631989 (PMC12557791; doi:10.3389/fvets.2025.1631989)
Supplement: Supplementary file 3 [file Data_Sheet_3.PDF]

Questionnaire 3 – PROSIM-DOG validation

This questionnaire forms part of a research study conducted by Professors Elvira Matilla Pinto and Carolina Balão da Silva, affiliated with the Biosciences Superior School of Elvas – Portalegre Polytechnic University. The purpose of this study is to evaluate the perceptions of Clinical and Academic Staff regarding the use of the PROSIM-DOG prostatic simulator for student academic training

Data collected will be used exclusively for academic and scientific purposes. Participant anonymity and data confidentiality will be strictly maintained in accordance with ethical research guidelines. No personally identifiable information will be recorded or disclosed.

There are no correct or incorrect responses. Participants are encouraged to respond sincerely and spontaneously to each item by marking an "X" in the appropriate box. Responses should reflect personal perceptions based on the use of the PROSIM-DOG simulator.

Thus, having been properly informed and clarified, I voluntarily agree to participate in this study/project. ☐

1. **How would you rate your overall experience with the prostatic palpation simulator?**
   ☐ Very good
   ☐ Good
   ☐ Fair
   ☐ Poor
   ☐ Very poor

2. **Do you consider the prostatic palpation simulator to be useful for learning the palpation technique?**
   ☐ Very useful
   ☐ Useful
   ☐ Neutral
   ☐ Slightly useful
   ☐ Not useful at all

3. **Does the simulator facilitate understanding of prostatic anatomy?**
   ☐ Yes, clearly and accurately
   ☐ Yes, but with some difficulties
   ☐ No, it is confusing
   ☐ No, the anatomy is unclear

4. **Is the texture of the simulator realistic and comparable to palpation in a live animal?**
   ☐ Very realistic
   ☐ Realistic
   ☐ Somewhat realistic
   ☐ Not very realistic
   ☐ Not realistic at all

5. **Does the simulator provide an adequate representation of both healthy and pathological prostates (e.g., tumors)?**
   ☐ Yes, completely
   ☐ Yes, with some shortcomings
   ☐ No, important details are missing
   ☐ No, it does not accurately represent pathological conditions

6. **How easy is it to use the simulator during practical sessions?**
   ☐ Very easy
   ☐ Easy
   ☐ Moderately easy
   ☐ Difficult
   ☐ Very difficult

7. **Do you believe the simulator helps improve students' skills in prostatic palpation?**
   ☐ Yes, significantly
   ☐ Yes, moderately
   ☐ No, only slightly
   ☐ No, it does not contribute to skill development

8. **What improvements would you suggest to make the simulator more effective as a teaching tool?**
   Open-ended response

9. **Do you have any additional comments or observations regarding the prostatic palpation simulator?**
   Open-ended response

Researchers greatly appreciate your participation. Your answers are essential for the continuous development of the simulator.
